# Luteolin decreases IGF-II production and downregulates insulin-like growth factor-I receptor signaling in HT-29 human colon cancer cells

**DOI:** 10.1186/1471-230X-12-9

**Published:** 2012-01-23

**Authors:** Do Young Lim, Han Jin Cho, Jongdai Kim, Chu Won Nho, Ki Won Lee, Jung Han Yoon Park

**Affiliations:** 1 Department of Food Science and Nutrition, Hallym University, Chuncheon, 200-702, Korea; 2 Medical & Bio-Materials Research Center, Kangwon National University, Chuncheon, 200-701, Korea; 3 Department of Food Science and Biotechnology, Kangwon National University, Chuncheon, 200-701, Korea; 4 Functional Food Center, Korea Institute of Science and Technology, Gangneung Institute, Gangneung, 210-340, Korea; 5 Department of Agricultural Biotechnology and Center for Agricultural Biomaterials, Seoul National University, Seoul, 151-921, Korea

## Abstract

**Background:**

Luteolin is a 3',4',5,7-tetrahydroxyflavone found in various fruits and vegetables. We have shown previously that luteolin reduces HT-29 cell growth by inducing apoptosis and cell cycle arrest. The objective of this study was to examine whether luteolin downregulates the insulin-like growth factor-I receptor (IGF-IR) signaling pathway in HT-29 cells.

**Methods:**

In order to assess the effects of luteolin and/or IGF-I on the IGF-IR signaling pathway, cells were cultured with or without 60 μmol/L luteolin and/or 10 nmol/L IGF-I. Cell proliferation, DNA synthesis, and IGF-IR mRNA levels were evaluated by a cell viability assay, [^3^H]thymidine incorporation assays, and real-time polymerase chain reaction, respectively. Western blot analyses, immunoprecipitation, and *in vitro *kinase assays were conducted to evaluate the secretion of IGF-II, the protein expression and activation of IGF-IR, and the association of the p85 subunit of phophatidylinositol-3 kinase (PI3K) with IGF-IR, the phosphorylation of Akt and extracellular signal-regulated kinase (ERK)1/2, and cell division cycle 25c (CDC25c), and PI3K activity.

**Results:**

Luteolin (0 - 60 μmol/L) dose-dependently reduced the IGF-II secretion of HT-29 cells. IGF-I stimulated HT-29 cell growth but did not abrogate luteolin-induced growth inhibition. Luteolin reduced the levels of the IGF-IR precursor protein and IGF-IR transcripts. Luteolin reduced the IGF-I-induced tyrosine phosphorylation of IGF-IR and the association of p85 with IGF-IR. Additionally, luteolin inhibited the activity of PI3K activity as well as the phosphorylation of Akt, ERK1/2, and CDC25c in the presence and absence of IGF-I stimulation.

**Conclusions:**

The present results demonstrate that luteolin downregulates the activation of the PI3K/Akt and ERK1/2 pathways via a reduction in IGF-IR signaling in HT-29 cells; this may be one of the mechanisms responsible for the observed luteolin-induced apoptosis and cell cycle arrest.

## Background

Colon cancer is the second most frequent cause of cancer-related death in the Western world [1]. Dietary patterns and lifestyle are the principal determining factors for colorectal cancer risk. The results of epidemiological studies have shown that the consumption of fruits and vegetables can reduce or prevent the risk of colon cancer [2]. Flavonoids are polyphenols, which are abundantly present in fruits and vegetables, and have been shown to have a variety of biological effects, including cancer prevention.

Insulin-like growth factors (IGFs) are polypeptides that stimulate the growth of a variety of mammalian cells [3]. These effects are mediated through the insulin-like growth factor I receptor (IGF-IR), and IGF-I and IGF-II are well-known ligands of IGF-IR. The binding of these ligands to IGF-IR results in the autophosphorylation of the receptor at the intracellular domain of β-subunits, resulting in the activation of the intrinsic tyrosine kinase of the IGF-IR. Subsequently, several adaptor molecules are recruited and activated via phosphorylation. Two distinct signaling pathways are activated by IGF-IR. The recruitment and activation of growth factor receptor-bound protein-2/son of sevenless or Shc can lead to the recruitment and activation of the Ras/Raf/mitogen activated protein kinase (MAPK) cascade, ultimately resulting in the activation of extracellular signal-regulated kinase (ERK)1/2. Alternatively, insulin receptor substrate-1 can be recruited and phosphorylated on multiple tyrosine residues that function as docking sites for the p85 subunit of phosphatidylinositol 3-kinase (PI3K) and activate the PI3K/Akt signaling pathway (reviewed in [4,5]). The activation of these pathways induces cell cycle progression and prevents apoptosis [6,7]. IGFs are also strong mitogens and survival factors for a variety of cancer cells, including prostate and colon cancer cells (Reviewed in [8]), and IGF-I and IGF-II mRNA levels were reported to be highly elevated in colon cancer [9]. We have previously reported that, in human colon cancer cells, including HT-29 cells and Caco-2 cells, IGF-II is synthesized and secreted, and an IGF-II autocrine loop stimulates the growth of these cancer cells [10,11].

Luteolin, 3',4',5,7-tetrahydroxyflavone, is found in a variety of vegetables, fruits, and medicinal herbs. Luteolin has been shown to function as an anti-oxidant, anti-inflammatory, and anti-cancer agent [12-15]. Additionally, luteolin induces cell cycle arrest and apoptosis in the liver and lung cancer and leukemia cell lines [16-20]. Our previous results indicated that luteolin inhibited HT-29 cell proliferation by inducing cell cycle arrest and apoptosis [21]. Therefore, in this study, we attempted to determine whether luteolin downregulates IGF-IR signaling in HT-29 cells.

## Methods

### Cell culture

Human colon cancer cells (HT-29 and Caco-2 cells) and rat intestinal epithelial cell line-6 (IEC-6 cells) were purchased from the American Type Culture Collection (Manassas, VA) and maintained in DMEM/F12 containing 100 mL/L of fetal bovine serum (FBS), with 100,000 U/L of penicillin and 100 mg/L of streptomycin. In order to determine the effects of luteolin and/or IGF-I on cell growth, we plated the cells with DMEM/F12 containing 10% FBS. Prior to luteolin treatment, the cell monolayers were subjected to 24 h of serum starvation with DMEM/F12 supplemented with 5 mg/L transferrin, 1 g/L BSA, and 5 μg/L selenium (serum-free medium). The cells were then incubated in serum-free medium with or without 60 μmol/L of luteolin (Sigma, St. Louis, MO, USA) and/or 10 nmol/L IGF-I (R & D System, Minneapolis, MN, USA) for 24, 48 or 72 h. Viable cell numbers were estimated via MTT assays. Luteolin was dissolved in DMSO and all cells were treated with DMSO at a final concentration of 0.1%.

### [^3^H]Thymidine incorporation assay

To determine DNA synthesis, the cells were plated at a density of 6,000 cells per well in 96-well plates and serum-starved as described above. After serum starvation, the cells were incubated for 2 h in serum-free medium containing 0 or 60 μmol/L of luteolin with or without IGF-I. 0.5 μCi [^3^H]thymidine was then added, and the incubation was continued for another 1 h. The incorporation of [^3^H]thymidine into DNA was estimated as previously described [11].

### IGF-II determination

For the determination of IGF-II, HT-29 cells were plated in 100 mm dishes at a concentration of 2 × 10^6 ^cells/dish and after 24 h, the monolayers were serum-starved and treated with various concentrations of luteolin (0 - 60 μmol/L) for 24 h. Conditioned media were collected and concentrated 20-fold, and immunoblot analysis was conducted using anti-IGF-II clone S1F2 (Upstate Biotechnology, Inc., Lake Placid, NY, USA) as previously described [22].

### Immunoprecipitation and immunoblot analyses

Cells were incubated for 2 h with 0 or 60 μmol/L of luteolin, and 10 nmol/L of IGF-I was added. At 0, 1, or 30 min after the addition of IGF-I, the cell lysates were prepared and immunoprecipitated with indicated antibodies. Immunoblot analyses were conducted as described previously [23]. Signals were detected via the enhanced chemiluminescence method using SuperSignal West Dura Extended Duration Substrate (Pierce, Rockford, IL, USA). The relative abundance of each protein band was analyzed via densitometric scanning of the exposed films. Immunoblots were probed with an antibody for β-actin as a protein loading control. The following antibodies were purchased from the indicated suppliers: anti-IGF-IRβ (C-20) (Santa Cruz Biotechnology, Inc., Santa Cruz, CA, USA); anti-phospho-tyrosine-RC20 antibody (PY20) linked to horseradish peroxidase (Transduction Laboratories, Palo Alto, CA, USA); anti-PI3K p85 antibody (Upstate Biotechnology, Inc.); anti-phospho-IGF-IR (P-IGF-IR, Abcam, Cambridge, MA, USA); and anti-ERK-1/2, anti-P-ERK-1/2 (Thr202/Tyr203), anti-cell division cycle 25c (CDC25c), anti-P-CDC25c, anti-Akt, and anti-P-Akt Ser473 (Cell Signaling Technology, Inc., Beverly, MA, USA).

### Real-time-polymerase chain reaction (RT-PCR)

Total RNA was isolated using RNeasy Plus Mini Kit (Qiagen, Valencia, CA, USA) and cDNA was synthesized using 3 μg of total RNA with SuperScript II reverse transcriptase (Invitrogen, Carlsbad, CA, USA). Real time-PCR was conducted as described previously [24]. Sequences used for primer sets were as follows: IGF-IR; forward-TGG AGT GCT GTA TGC CTC TG, backward-TGA TGA CCA GTG TTG GCT GG, β-actin; forward-GTT TGA GAC CTT CAA CAC CCC, backward-GTG GCC ATC TCC TGC TCG AAG TC. The levels of mRNA were normalized to β-actin and the control (0 μmol/L luteolin) levels were set to 100%.

### PI3K assay

PI3K activity was estimated as described previously [25]. Cell lysates (1 mg protein) were immunoprecipitated with a polyclonal antibody against IGF-IRβ followed by incubation with protein A-Sepharose beads. After washing, the beads were resuspended in 20 μL of kinase buffer containing 4 μg of phosphatidylinositol (Sigma, St. Louis, MO, USA), 10 μmol/L of ATP, 5 mmol/L of MnCl_2_, and 10 μCi of [γ-^32^P]ATP and incubated for 20 min at 30°C. In order to determine whether luteolin directly inhibits the kinase activity of PI3K, active PI3Kα (100 ng, Millipore, Billerica, MA, USA) was incubated for 10 min in the absence or presence of 20 μmol/L of luteolin at 30°C in 20 μL of kinase buffer. Phosphatidylinositol (25 μg) was added and the incubation was continued for another 5 min at room temperature. 10 μCi of [γ-^32^P]ATP was then added and reactions were incubated for 10 min at 30°C. The resultant ^32^P-labeled phosphatidylinositol 3-phosphate (PIP) lipids were separated from reaction products by thin layer chromatography (TLC) and visualized by autoradiography. The radioactive PIP signals were quantitated via densitometry using the Bio-profile Bio-1D application (Vilber-Lourmat, France) [23].

### Statistical analyses

Data were expressed as means ± SEM values and analyzed via analysis of variance. Differences between treatment groups were analyzed by Duncan's multiple range test or Student's t-test. The means were considered significantly different at *P *< 0.05. All statistical analyses were conducted using the SAS system for Windows, version 8.12 (SAS, Inc., Cary, NC, USA).

## Results

### Luteolin reduces IGF-II secretion in HT-29 cells

**Figure 1:**
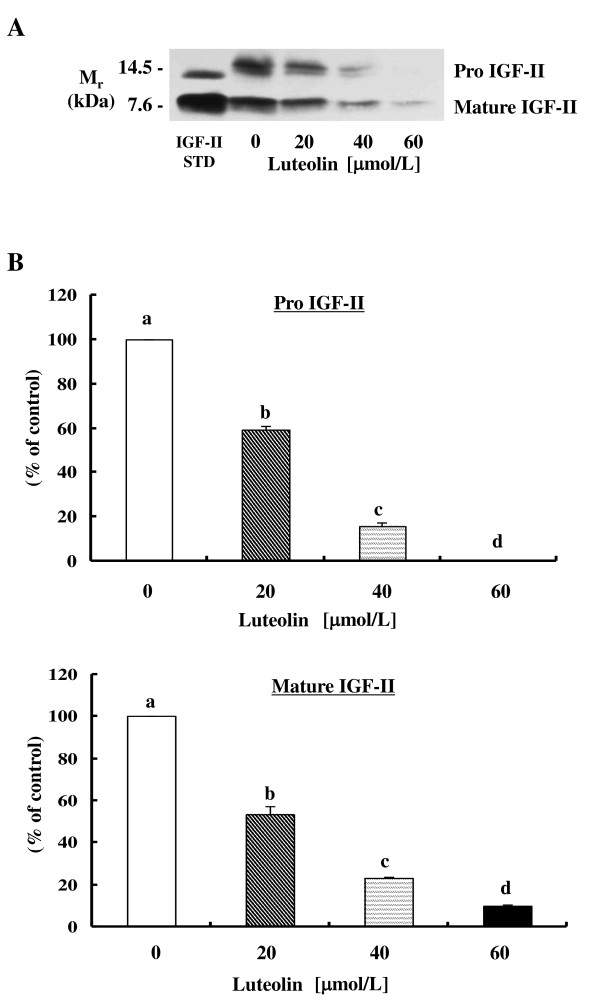
**Luteolin reduces IGF-II secretion dose-dependently in HT-29 human colon cancer cells**. (**A**) HT-29 cells were plated at a density of 2 × 10^6 ^cells/100 mm dish in DMEM/F12 supplemented with 10% FBS. After 24 h, cells were serum-starved with serum-free DMEM/F12 supplemented with 5 mg/L of transferrin and 5 μg/L of selenium for 24 h. Cells were treated with various concentrations (0 - 60 μmol/L) of luteolin. 24 h after luteolin treatment, conditioned media were collected and concentrated for immunoblot analysis with an anti-IGF-II antibody. The volumes of media loaded onto the gels were adjusted for equivalent cell numbers. Photographs of the chemiluminescent detection of the blot, which were representative of three independent experiments, are shown. (**B**) The relative abundance of each band was quantified via densitometric scanning of the exposed films, and the control levels were set at 100%. Each bar represents the mean ± SEM (n = 3). Means without a common letter differ, *P *< 0.05.

In the previous study, we observed that luteolin inhibited HT-29 human colon cancer cell proliferation by inducing cell cycle arrest and apoptosis [21]. However, the treatment of IEC-6 rat intestinal epithelial cells at the same concentrations (20 - 60 μmol/L) of luteolin for 24 h did not alter the viability of these cells (data not shown). In order to assess the effect of luteolin on IGF-II secretion of HT-29 cells, cells were treated with 20 - 60 μmol/L of luteolin for 24 h and conditioned media were assayed via immunoblot analysis. Luteolin reduced the secretion of pro- and mature-IGF-II in a dose-dependent manner (Figure 1).

### Luteolin abrogates the growth stimulatory effects of exogenous IGF-I on HT-29 cells

**Figure 2:**
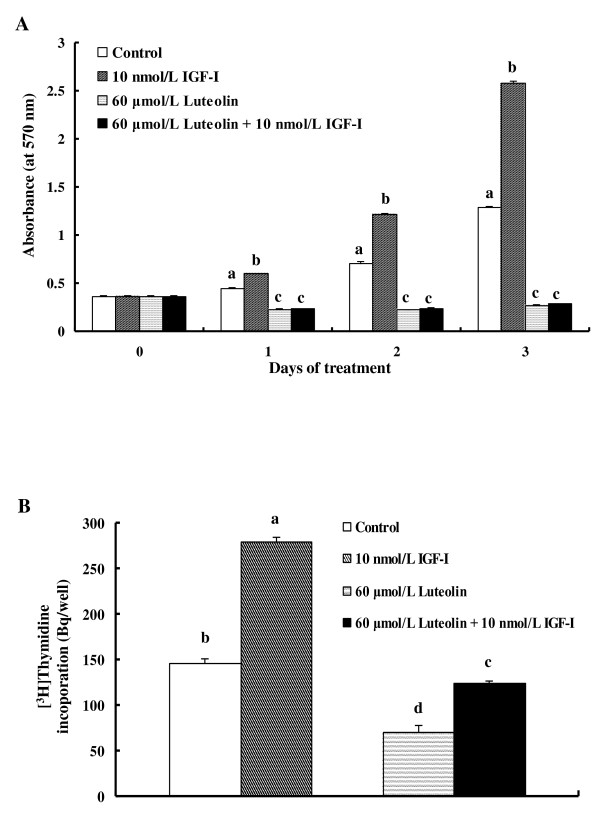
**Luteolin abrogates the growth-stimulatory effects of exogenous IGF-I in HT-29 cells**. (**A**) HT-29 cells were plated in 24-well plates at a density of 5 × 10^4 ^cells/well. One day later, the cells were serum-starved for 24 h with serum-free DMEM/F12 supplemented with 5 mg/L transferrin, 0.1 g/L BSA, and 5 μg/L selenium for 24 h. After serum-starvation, the cells were incubated in serum-free medium containing 0 or 60 μmol/L of luteolin with or without 10 nmol/L of IGF-I for 24, 48, and 72 h. Viable cell numbers were estimated via an MTT assay. (**B**) Cells were plated in 96-well plates at a density of 6 × 10^3 ^cells/well. Cells were serum-starved and then treated with 0 or 60 μmol/L of luteolin with or without 10 nmol/L of IGF-I for 2 h. [^3^H]Thymidine was then added, and the incubation was continued for an additional 1 h in order to measure its incorporation into DNA. Each bar represents the mean ± SEM (n = 6). Means without a common letter differ, *P *< 0.05.

In order to determine whether luteolin inhibits the growth-stimulatory effects of exogenous IGF-I, cells were treated with 0 or 60 μmol/L of luteolin in the absence or presence of 10 nmol/L IGF-I for 24, 48, or 72 h. IGF-I increased but luteolin significantly reduced the numbers of viable cells. The treatment of cells with IGF-I did not alleviate the growth-inhibitory effects of luteolin (Figure 2A). To explore the effect of luteolin on DNA synthesis in HT-29 cells, an [^3^H]thymidine incorporation assay was conducted. DNA synthesis was induced by IGF-I treatment, and luteolin significantly inhibited the stimulatory effect of IGF-I (Figure 2B).

### Luteolin reduces the levels of the IGF-IR precursor protein and IGF-IR transcripts in HT-29 cells

**Figure 3:**
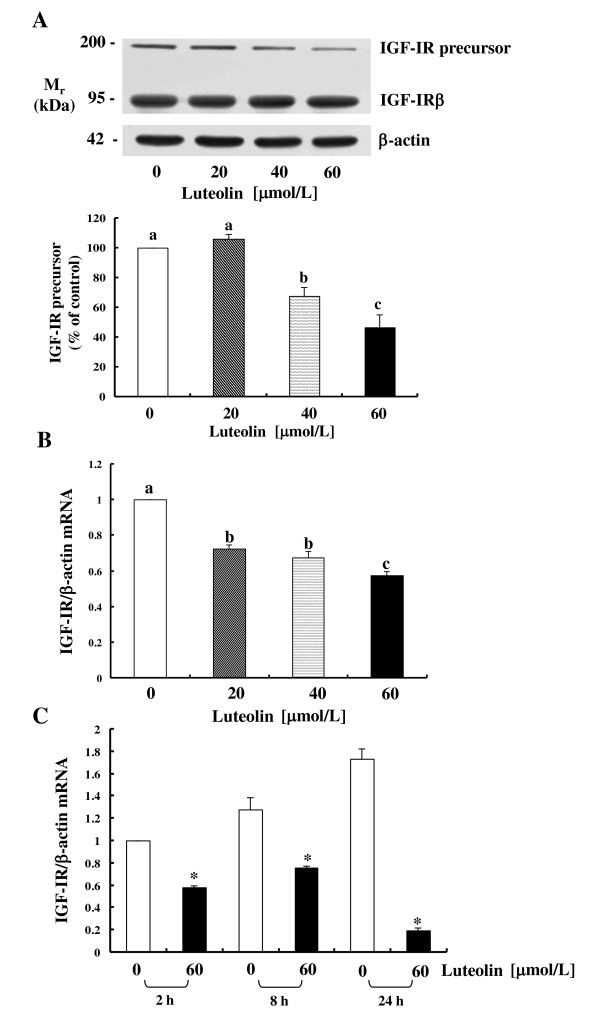
**Luteolin reduces the levels of the IGF-IR protein and mRNA in HT-29 cells**. (**A**) HT-29 cells were plated and treated with luteolin as described in Figure 1 for 2 h. Total cell lysates were prepared and immunoblot analyses were conducted. Photographs of the chemiluminescent detection of the blots, which were representative of three independent experiments, were shown. The relative abundance of IGF-IR to their own β-actin was quantified via densitometric scanning of the exposed films, and the control levels were set at 100%. (**B**)HT-29 cells were plated and treated with luteolin as described in Figure 1 for 2 h. (**C**)HT-29 cells were treated with 0 or 60 μmol/L of luteolin for 2, 8, and 24 h. (**B, C**) Total RNA was isolated and real-time PCR was conducted. Each bar represents mean ± SEM (n = 3). (**A, B**) Means without a common letter differ, *P *< 0.05. (**C**) *Different from 0 μmol/L of luteolin at each treatment time, *P *< 0.05.

Because luteolin reduced IGF-II secretion but exogenous IGF-I did not abrogate the growth-inhibitory effect of luteolin, we attempted to determine whether luteolin inhibits the IGF-I signaling pathway. Western blot analysis revealed that the levels of the IGF-IR precursor protein were reduced 2 h after the addition of luteolin, whereas the levels of the IGF-IR β-subunit were unaltered (Figure 3A). Additionally, IGF-IR transcript levels were reduced dose-dependently in cells treated with luteolin for 2 h (Figure 3B). Furthermore, the levels of IGF-IR mRNA were reduced further at 24 h of luteolin treatment as compared to those at 2 h (Figure 3C).

### Luteolin inhibits IGF-I-induced activation of IGF-IR, Akt, and ERK1/2 in HT-29 cells

In order to determine whether luteolin down-regulates IGF-I-induced tyrosine phosphorylation of the IGF-IR, cells were treated for 2 h with 0 or 60 μmol/L of luteolin, and IGF-IR was stimulated with 10 nmol/L IGF-I for 0, 1, or 30 minutes. Total cell lysates were prepared and immunoprecipitated using an IGF-IRβ antibody. The immune complexes were used for Western blot analysis with an anti-P-tyrosine antibody (PY20). IGF-I induced tyrosine phosphorylation of IGF-IR at 1 min; tyrosine phosphorylation levels were slightly reduced at 30 min. Luteolin significantly inhibited the phosphorylation of IGF-IRβ at 1 min after IGF-I treatment. At 30 min, the phosphorylation status of IGF-IR did not differ between the control and luteolin-treated cells.

**Figure 4:**
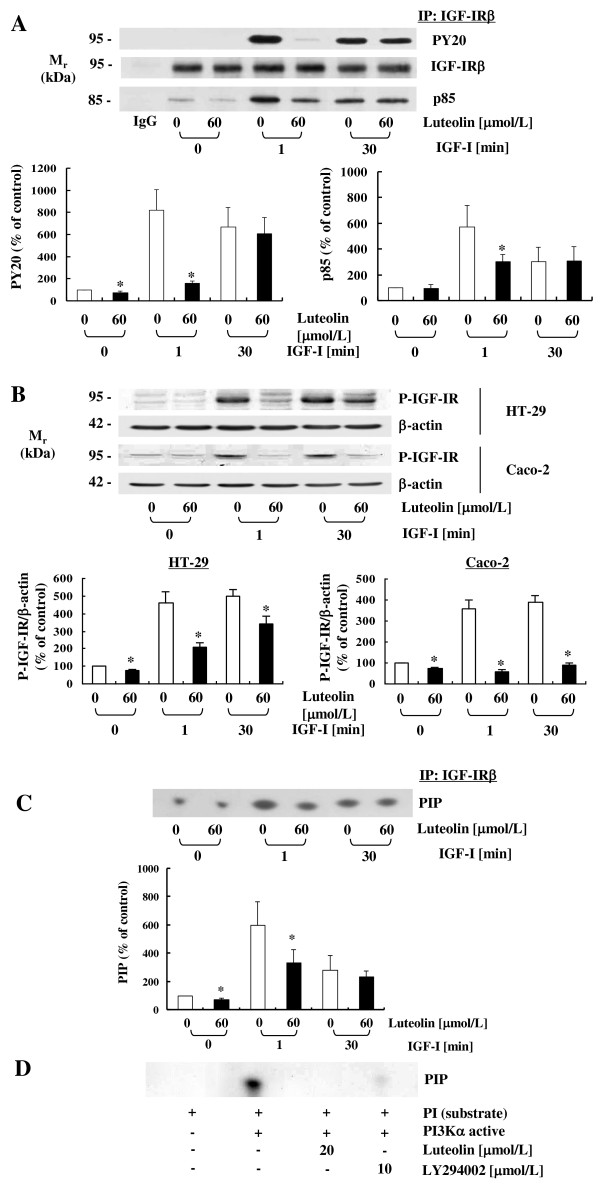
**Effects of luteolin on IGF-I-induced tyrosine phosphorylation of IGF-IR, the association of p85 with IGF-IR, and PI3K activity in human colon cancer cells**. Cells were plated and cultured as described in Figure 1. (**A**) HT-29 cells were treated for 2 h with 0 or 60 μmol/L of luteolin and lysed with or without stimulation of 10 nmol/L IGF-I for 0, 1, or 30 minutes. Total cell lysates were incubated with anti-IGF-IRβ antibody and the immune complexes were precipitated with protein A-Sepharose. The immunoprecipitated proteins were analyzed via Western blotting with antibodies raised against phosphotyrosine (PY20), IGF-IRβ, or p85. (**B**) HT-29 and Caco-2 cells were plated and treated as described above. Total cell lysates were analyzed via Western blotting with an antibody raised against P-IGF-IR. Photographs of the chemiluminescent detection of the blots, which were representative of three independent experiments, were shown. (**C**) The immune complexes obtained from HT-29 cells were incubated with phosphatidylinositol and [γ-^32^P]ATP. (**D**) Active PI3K and luteolin were incubated with phosphatidylinositol and [γ-^32^P]ATP as described in the Materials and Methods section. Phosphatidylinositol 3-phosphate (PIP) generated by immunoprecipitated PI3K (**C**) or active PI3Kα (**D**) was separated via thin-layer chromatography (TLC). An autoradiograph of the TLC plate, which was representative of three independent experiments, is shown. (**A, B, C**) The relative abundance of each blot was quantified via densitometric scanning of the exposed films and the control levels (0 μmol/L luteolin, without IGF-I stimulation) were set at 100%. Each bar represents the mean ± SEM (n = 3). *Different from 0 μmol/L of luteolin at a stimulation time, *P *< 0.05.

**Figure 5:**
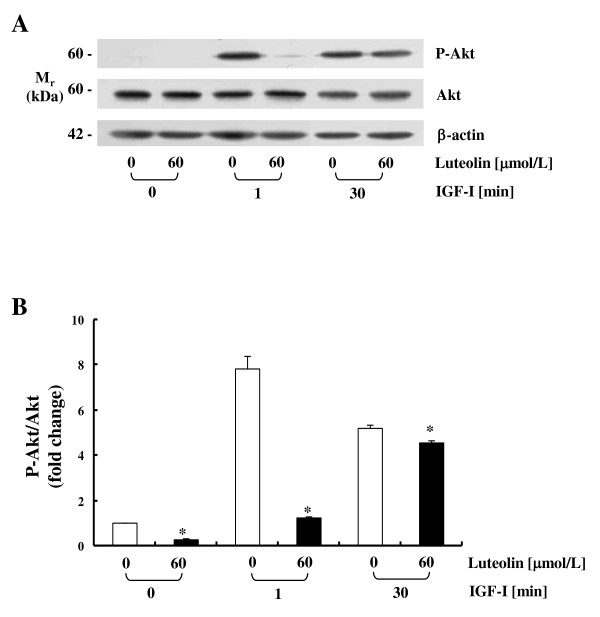
**Luteolin inhibits IGF-I-induced Akt activation in HT-29 cells**. Cells were plated and treated as described in Figure 4. Total lysates were prepared and immunoblot analyses were conducted with antibodies raised against p-Akt, Akt, or β-actin. (**A**) Photographs of the chemiluminescent detection of the blots, which were representative of three independent experiments, were shown. (**B**) The levels of P-Akt to its own Akt control band on immunoblots were quantified via densitometric scanning of the exposed films, and the control levels (0 μmol/L luteolin, without IGF-I stimulation) were set at 1. Each bar represents the mean ± SEM (n = 3). *Different from 0 μmol/L of luteolin at a stimulation time, *P *< 0.05.

To evaluate the association of the p85 subunit of PI3K with IGF-IR, we conducted immunoprecipitation of cell lysates with an IGF-IRβ antibody and subsequent immunoblotting with a p85 antibody. IGF-I stimulated the association of the p85 regulatory subunit of PI3K with IGF-IR within 1 min, which was significantly inhibited by luteolin treatment (Figure 4A). The association of p85 with IGF-IR was reduced at 30 min, and no difference was observed in the association of these two molecules between the control and luteolin-treated cells at this time period. Western blot analysis of total cell lysates revealed that IGF-I markedly increased P-IGF-IR levels and luteolin reduced those in both HT-29 and Caco-2 cells (Figure 4B). For the determination of PI3K activity, the immune complex was incubated with [^32^P]ATP and phosphatidylinositol. Luteolin reduced both basal and IGF-I-induced PI3K activity in HT-29 cells at 1 min of IGF-I treatment. However, this difference disappeared at 30 min after IGF-I treatment (Figure 4C). In order to determine whether the luteolin-induced inhibition of PI3K is the result of direct interaction with this kinase, active PI3K was incubated with 20 μmol/L of luteolin in the kinase reaction. Luteolin inhibited PI3K activity in a cell-free system (Figure 4D). The activation of PI3K leads to the activation of Akt [7]. Akt phosphorylation was induced by IGF-I treatment at 1 min without any changes in total Akt expression; luteolin significantly reduced the level of Akt activation (Figure 5).

**Figure 6:**
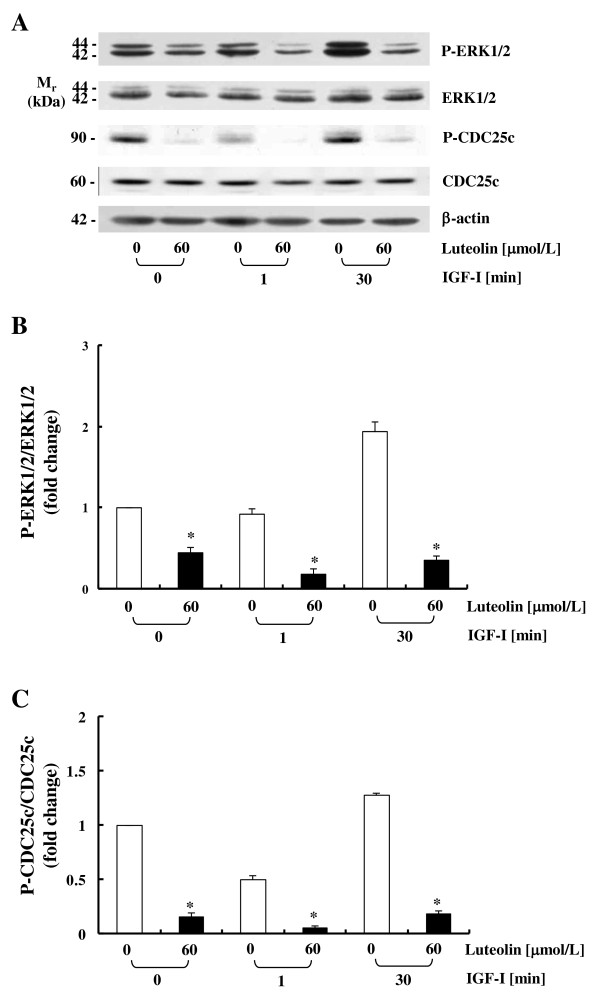
**Luteolin inhibits IGF-I-induced ERK1/2 and CDC25c activation in HT-29 cells**. Cells were plated and treated as described in Figure 4. Total lysates were prepared and immunoblot analyses were conducted with antibodies raised against P-ERK1/2, ERK1/2, P-CDC25c, CDC25c, or β-actin. (**A**) Photographs of the chemiluminescent detection of the blots, which were representative of three independent experiments, were shown. (**B, C**) The levels of P-ERK1/2 to its own ERK1/2 control band (**B**) and those of P-CDC25c to its own CDC25c control band (**C**) on immunoblots were quantified via densitometric scanning of the exposed films, and the control levels (0 μmol/L luteolin, without IGF-I stimulation) were set at 1. Each bar represents the mean ± SEM (n = 3). *Different from 0 μmol/L of luteolin at a stimulation time, *P *< 0.05.

IGF-I stimulated ERK1/2 activation in HT-29 cells was detected at 30 min of IGF-I treatment, and luteolin inhibited the phosphorylation of ERK1/2 in the absence or presence of IGF-I treatment (Figure 6). Because ERK1/2 activation was reported to lead to the activation of the protein phosphatase CDC25c during the G2/M transition of cell cycle progression [26], we subsequently attempted to determine whether luteolin treatment results in a reduction in the phosphorylation of CDC25c. The levels of P-CDC25c were increased at 30 min after IGF-I addition and significantly reduced in cells treated with luteolin, regardless of whether or not the HT-29 cells were treated with IGF-I (Figure 6C).

## Discussion

The IGF system (IGF-I, IGF-II, IGF-binding protein, and IGF-IR) performs an important role in the growth of various cancer cells, including colon cancer cells [8,27]. We have reported previously that luteolin inhibited the proliferation of HT-29 human colon cancer cells by inducing cell cycle arrest and apoptosis [21]. The results of a previous study revealed that luteolin reduced the expression of cyclin D1 and cyclin B1 and inhibited the activities of CDKs, thereby suppressing HT-29 cell cycle progression. Additionally, luteolin induced the activation of caspases and reduced the levels of proteins involved in the suppression of apoptosis, including Bcl-xL and Mdm-2 [21]. Thus, in the present study, we explored the upstream signals that are important for the regulation of cell cycle progression and apoptosis in HT-29 cells. Our previous data demonstrated that HT-29 cells synthesized and secreted IGF-II and expressed IGF-IR, and that IGF-II stimulated HT-29 cell growth via an autocrine mechanism [10,28]. Kim *et al. *also reported that the reduction of IGF-II secretion in Caco-2 colon cancer cells inhibited cell growth [11]. Using PC-3 and DU145 human prostate cancer cells, Fang et al. [29] have demonstrated that luteolin inhibits the IGF-I-induced activation of IGF-IR and AKT as well as the downstream targets of AKT, p70S6K1, GSK-3β, and FKHR/FKHRL1. In the present study, we demonstrate that, in HT-29 human colon carcinoma cells, luteolin 1) reduces IGF-II secretion; 2) inhibits the growth-stimulatory effects of IGF-I; 3) reduces the levels of IGF-IR transcripts and the IGF-IR precursor protein; 4) reduces the IGF-I-induced tyrosine phosphorylation of IGF-IRβ and the association of p85 with IGF-IRβ; 5) inhibits IGF-I-induced PI3K activity 6) inhibits IGF-I-induced Akt activation; and 7) inhibits the IGF-I-induced phosphorylation of ERK1/2 and CDC25c. These results indicate that the reduction in IGF-II secretion and changes in IGF-IR signaling by luteolin may be important factors underlying the growth-inhibitory effects of HT-29 cells. Additionally, we have demonstrated that luteolin directly inhibits the activity of PI3K in a cell-free system.

When HT-29 cells were treated with exogenous IGF-I, IGF-I did not abrogate the growth-inhibitory effects of luteolin (Figure 2), although luteolin reduced IGF-II secretion (Figure 1). These results indicated that luteolin inhibits IGF-IR signaling in HT-29 cells. IGF-IR consists of two extracellular α-subunits and two transmembrane β-subunits, and IGF-I and IGF-II bind to the α-subunits of IGF-IR, thus resulting in the activation of the intrinsic tyrosine kinase in the intracellular domain of the β-subunits [28]. In this study, luteolin reduced the levels of the IGF-IR precursor but did not reduce the levels of IGF-IR β-subunits; this suggests that the levels of IGF-IR α-subunits may have been reduced by luteolin treatment. The finding that IGF-IR mRNA levels were continuously decreased during 24 h of luteolin treatment (Figure 3C) indicates that the expression of IGF-IR protein is regulated by luteolin, at least in part, at an RNA level. The effects of luteolin on IGF-IR mRNA and protein stability will require further study in the future.

Fang *et al. *demonstrated that prostate cancer cells in which the IGF-IR gene is knocked down grew at a slower rate relative to that in control cells, and the inhibition of cell growth by luteolin treatment was similar to that observed in IGF-IR-depleted cells [29]. In this study, we demonstrate that luteolin inhibits IGF-II secretion, and that IGF-I-stimulated HT-29 cell proliferation was inhibited by luteolin (Figure 2). These results suggest that the inhibition of the IGF/IGF-IR signaling pathway by luteolin might be one of the mechanisms for the suppression of proliferation and apoptosis in HT-29 cells. In 1994, Lahm et al. demonstrated that Alpha IR3, a neutralizing monoclonal antibody directed against human IGF-IR, inhibited proliferation in HT-29 cells [30]. It has also been demonstrated that the blockade of IGF-IR with IGF-IR monoclonal antibodies inhibited proliferation, arresting the cell cycle and inducing the apoptosis of HT-29 cells [31]. Additionally, an anti-human/mouse IGF-II-neutralizing antibody effectively inhibited the hepatic metastasis of HT-29 cells [32]. *In vitro *experiments have also demonstrated that IGF-II-neutralizing antibody treatment completely blocked IGF-IR phosphorylation in serum-starved HT-29 cells [33]. These results indicate that IGF-II is an autocrine growth factor of HT-29 cells and that the inhibition of IGF-II secretion and/or IGF-IR signaling inhibits HT-29 cell proliferation.

In our HT-29 cells, it is possible that the luteolin-induced downregulation of the IGF-IR α-subunit results in reduced phosphorylation of the β-subunit. It is also possible that luteolin directly interferes with the binding of IGF-I to IGF-IR, which would consequently inhibit the phosphorylation of the β-subunit. This reduced IGF-I-induced tyrosine phosphorylation of IGF-IRβ by luteolin led to the reduced association of p85 with IGF-IRβ and the subsequent activation of PI3K/Akt and ERK1/2 (Figures 4, 5 and 6). Additionally, luteolin inhibited PI3K activity in a cell-free system (Figure 4D), thereby indicating that luteolin can also modulate the activity of this enzyme via direct interaction with this kinase. As the activation of Akt and ERK1/2 induces cell proliferation and inhibits apoptosis in various cancers [34,35], the PI3K/Akt and ERK1/2 pathways may be important targets in cancer therapies involving natural bioactive compounds [6,23,28,29,36-38]. Akt regulates the expression and activity of proteins involved in the regulation of apoptosis and cell cycle progression, including Bad, p21, cyclin D1, and Mdm-2 (Reviewed in [37]). Previously, we have demonstrated that luteolin downregulates the expression of Mdm-2 and cyclin D1 [21]. Fang *et al. *also reported that luteolin treatment induced a reduction in the levels of P-IGF-IR, P-Akt, and cyclin D1 in PC3 prostate cancer cells [29]. The results of previous studies and of the present study indicate that the inhibition of Akt activation by luteolin may result in the downregulation of Mdm-2 and cyclin D1, which may contribute to the induction of apoptosis and cell cycle arrest in colon and prostate cancer cells. Collectively, these results indicate that the downregulation of IGF-IR/PI3K/Akt by luteolin is one of the principal signaling pathways for the induction of cell cycle arrest and apoptosis in HT-29 cells.

ERK-MAP kinases also regulate cell cycle- and apoptosis-related proteins. ERK1/2 activation leads to the phosphorylation of the protein phosphatase CDC25c during the G2/M transition of cell cycle progression [26]. Phosphorylated CDC25c dephosphorylates CDC2, which results in the activation of the CDC2/cyclin B1 complex. Luteolin has been reported to reduce the levels of the CDC25c, CDC2, and cyclin B1 proteins and induces G2/M phase arrest in human gastric cancer cell lines [39]. In our previous study, luteolin reduced cyclin B1 levels, markedly inhibited CDC2 activity, and promoted G2/M phase arrest in HT-29 cells [21]. In the present study, we determined that luteolin reduced the levels of P-CDC25c in HT-29 cells (Figure 6C). Together, these results indicate that the attenuated ERK1/2 activation contributed to the reduction of P-CDC25c levels in luteolin-treated cells. The reduction in CDC25c activation may have contributed to the induction of G2/M arrest in HT-29 cells.

## Conclusions

**Figure 7:**
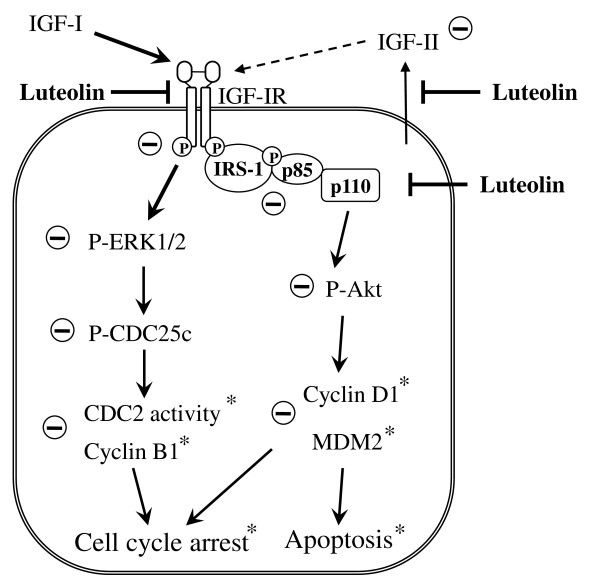
**A tentative scheme for luteolin regulation of the IGF-IR signaling pathway in HT-29 human colon cancer cells**. Luteolin reduces the secretion of IGF-II and levels of IGF-IR mRNA and protein, which leads to a reduction in IGF-IR phosphorylation and a subsequent inhibition of PI3K/Akt and ERK1/2/CDC25c activation. Additionally, luteolin directly inhibits PI3K activity. These changes in IGF-I signaling contribute to luteolin-induced apoptosis and cell cycle arrest in HT-29 cells. *From our previously published results [21].

We demonstrated that luteolin reduced the secretion of pro- and mature-IGF-II and reduced the levels of the IGF-IR precursor protein in HT-29 cells, and subsequently reduced the activation of the Akt and ERK1/2 pathways. The inhibition of the IGF-IR signaling pathway may be one of the mechanisms by which luteolin inhibits Akt and ERK1/2 signaling in HT-29 cells, thereby inhibiting cell growth and inducing apoptosis (Figure 7). The present results help delineate the mechanisms of luteolin actions for future animal studies with colon cancer models. Such studies will determine whether luteolin can be developed into a chemopreventive agent for use against colon cancer.

## Abbreviations

IGF-IR: insulin-like growth factor-1 receptor; IGF-II: insulin-like growth factor-II; ERK-1/2: extracellular signal-regulated kinase-1/2; PI3K: phophatidylinositol-3 kinase; PIP: phosphatidylinositol 3-phosphate; TLC: thin layer chromatography; CDK: cyclin-dependent kinase; CDC: cell division cycle.

## Competing interests

The authors declare that they have no competing interests.

## Authors' contributions

DYL, JK, KWL, and JHYP planned and designed this research; DYL and HJC performed the assays and analyzed the data; DYL wrote the first draft and JHYP, DYL, HJC, and CWN revised the paper. All authors read and approved the final manuscript.

## Pre-publication history

The pre-publication history for this paper can be accessed here:

http://www.biomedcentral.com/1471-230X/12/9/prepub
